# Letter from H. Wardner, Surgeon, 12th Ill. Vols.

**Published:** 1862-05

**Authors:** 


### Army Correspondence.

PITTSBURG LANDING, TENN., *April* 17, 1862.

EDITORS MEDICAL EXAMINER:

Desirous of adding field service to my experience in military surgery, I have, as you know, accepted a position in the army. I chose the artillery in preference to infantry, or brigade service, because that arm of service is always in action, and gives the greatest variety of gunshot injuries. Accordingly, I have pitched my tent under these greenwood trees, as the surgeon of the 1st Regiment of Ill. Light Artillery, Col. Webster commanding.

Many volumes might be written on the surgery of this great battle of Pittsburg Landing, but I will only give you a cursory sketch of such points as are at present most easily ascertainable. The wounds were of every conceivable character, from a scratch of the epidermis to the knocking off the shoulders of the whole head.

I saw several exceptions to the general rule, that the modern elongated bullets keep a straight course, not turning to the right or left for any obstacle. I cut out one from the thigh, which had glanced from the femur. It was bruised out of shape, and flew in a crooked course, beneath the skin, to a distant point in the limb, while the bone was not broken. Other similar cases were occasionally to be found. I cut out several balls from beneath the scalp, which had flattened themselves to wafers against the skull, without breaking the bone. Several cases fell under my notice, in which balls barely penetrated the outer clothing and stopped, merely brusing the skin beneath.

The projectiles of the enemy were round shot, shell, cannister, grape, long bullets, round bullets, and buckshot. There seemed to me to be an unusual number of wounds from spent balls, which was explained when I saw a captured box of rebel cartridges. Many of these only contained half the usual charge of powder, apparently being thus made from motives of economy of that material. The slaughter, however, was terrible. Our wounded must number from three to five thousand. The surgeons showed commendable courage, and, indeed, seem to have exposed their lives unjustifiably, in some cases. One surgeon, whose name I cannot learn, was killed, and six or seven were wounded. Among the latter, was Dr. Frank Reilly, the junior editor of your Journal. He was shot in the leg, fracturing the fibula, while attending to the wounded of the Illinois Lead Mine Regiment, as assistant-surgeon. His wound disabled him from field service, and necessitated sending him home for recovery. Dr. Roskotten, of Peoria, was injured. His horse was shot under him, and falling on his leg, disabled him from field service. He went on board a hospital steamer, and rendered valuable service among the wounded there.

Dr. H. Wardner, late demonstrator of anatomy, in the Medical Department of Lind University, was very active, and has furnished me the following account of his operations:—

Dr. Andrews, *Dear Sir:*—On the 6th inst. our regiment was ordered into battle, on the extreme left of our lines, to support Col. Stuart's brigade. I followed the regiment with my hospital attendants and regimental brass band, taking with me a supply of dressings for temporary use on field. While seeking a suitable position for a field depot, I came within direct range of one of the enemy's batteries. I ordered my attendants to lie down behind a small elevation of ground, until the range might be changed, which was done in about fifteen minutes. I then, with assistant-surgeon Bane, of the 50th Illinois, established a depot in a ravine, about three hundred yards in rear of our line of battle.

At this plrce, *ten* of my regiment were brought to me, two mortally, and the rest badly wounded; among the latter Capt.

Swain. At this time, the balls, shells, and bullets came in such volleys over our heads, that we knew the enemy to be rapidly advancing upon the ground, and about ten minutes later, had the dissatisfaction of seeing, from an opposite bluff, our depot ground in possession of the enemy. I then moved towards the Landing, and met Dr. Hewitt, medical director, at the log building used as post office, and received an order from him to give my attention to the wounded in the tents near the steamer City of Memphis. At this place, I had amputated one arm, and had just taken the knife to operate upon another, when a rifle ball whistled over our heads, doubtless fired at the men at a battery about fifty yards in our front.

The battery then opened upon the enemy and thus drew their fire upon us. All the wounded who could crawl out of their tents were immediately seen scrambling down the river bank, some upon their haunches, others upon their bellies, and in all shapes were seeking a cover from the danger threatening them. Night soon put an end to our efforts in surgery, and taking a few of the worst cases about me, I went on board the steamer Chessman, and remained until the afternoon of Monday.

On board this boat I performed five amputations. On the morning of Tuesday, I was ordered to report to Brigade-surgeon Derby, on the steamer City of Memphis, and was by him placed in charge of the wounded on the hurricane deck. On this day, I performed five capital operations, assisted by Dr. Shirriff, and an invalid surgeon, whose name I disremember.

On Monday morning, the boat went down the river, and I rejoined my regiment, where I performed one amputation.

On Sunday the 13th, I succeeded in getting the last of our seriously wounded in hospital boats.

My regiment lost 22 killed, and 86 wounded.

The following is a table of operations, with results as far as known:—

| | | | | | |
|---|---|---|---|---|---|
| 1. | Left arm, | Amputated | above the | elbow, | Successful. |
| 2. | Right " | " | " | " | " |
| 3. | " " | " | " | " | " |

| | | | | |
|---|---|---|---|---|
| 4. | Right leg, | Amputated, | upper third, | Successful. |
| 5. | " thigh, | " | middle " | " |
| 6. | " leg, | " | upper " | " |
| 7. | Left thigh, | " | " " | Fatal. |
| 8. | Right leg, | " | " " | Successful. |
| 9. | " arm, | " | " " | " |
| 10. | " thigh, | " | middle " | Fatal. |
| 11. | " leg, | " | upper " | Successful. |

Besides the above, I performed many minor operations, as amputations of fingers, extractions of balls, &c., &c.

It may be interesting to append the following list of amputations, &c., performed by me at Fort Donelson:—

| | | | |
|---|---|---|---|
| 1. | Fore Arm, | Middle third. | Successful. |
| 2. | Arm, | " " | " |
| 3. | Right leg, | " " | " |
| 4. | " thigh, | Lower " | " |
| 5. | Left shoulder, | At joint, | Fatal in 5 weeks. |

6. Lower jaw, large portion removed leaving angle and condyles. Successful.

7. Extraction of a grape-shot.

This entered the right cheek, in front of the angle of the lower jaw, knocking out the back molars, passed into and through the pharynx, opening the trachea, (so that when the man tried to drink he was strangled,) and lodged on the left side of the neck, resting upon the brachial plexus of nerves, thus causing paralysis of the left arm. I removed the ball without the loss of more that a drachm of blood. Brigade-surgeon Brenton being in consultation.

Several surgeons have since claimed the credit of this peculiar operation.

The extraction was performed by cutting at the outer border of the mastoid muscle, pushing the external jugular vein back, and the muscle forward. The dissection was then continued with the handle of the scalpel, and the ball raised out with an elevator.

H. WARDNER, Surgeon, 12th Ill. Vols.

I saw one instance showing how an operation seems to stimulate and revive a sinking patient. I found a poor "secesh" fellow on the wet ground in a tent, three days after the battle, with a minnie ball hole through, shattering and comminuting, the femur, and another similar one through the tibia. Gangrene had already set in, the pulse could hardly be felt at the wrist, and the case seemed hopeless. Giving him what attention I could at the moment, I went on to assist others, but after an hour or two I returned to him and determined to give him the chance of an amputation. Accordingly, I put him under the influence of chloroform, when the last remnant of pulse disappeared from the wrist. I, however, proceeded and amputated the thigh at the middle third. As I proceeded the pulse returned, and in an hour he was better than he had been for twenty-four hours. He was put on stimulants and the next day was still further improved. He was then sent to a hospital steamer, and I was unable to learn his fate.

The system of management here is most advantageous for the wounded; but it is unfortunate that no reliable statistics can ever result from it. The wounded are rapidly dressed on the field and some amputations, etc., there performed. The pressure of the labor, however, on the surgeon, prevents any adequate written account of all his cases, and never allows him to preserve full record of names, companies, and regiments. From the hands of the field-surgeons, the patients are taken by ambulances to hospital steamers, where any necessary additional operations and dressings are made. As the number of surgeons on board is very limited, they are taxed to the utmost, and have no time for recording their cases. As soon as the boat is filled she moves down the river to Paducah, Mound City or, other general hospitals, and discharges her suffering cargo. Here the secondary operations are performed, and here only, are any perfect records possible. The field-surgeon may remember and write down many cases, but he cannot ascertain their subsequent history. The boat-surgeon knows neither the subsequent nor previous condition; and the general hospital-surgeon knows nothing of the previous history, nor has he any account from those who have preceded him. For these reasons the surgical history of this war will never be perfectly written.

The Government supplies of medicines here are very inadequate to the necessities of the case. I have not been able to get anything at all aproximating to the field supply table. For a considerable time I could not procure a single article of the mercurial class, and the third day after the battle I drew the last half-pound of chloroform but one, from the purveyor. Fortunately, I obtained a new stock of the latter from the Sanitary Commission stores. I have not been able to procure any veratrum viride, nor a single expectorant, except buchu, to this day. This dearth is partly owing to losses of regimental stores in the battle.

Some of the surgeons here are, as might be expected among so many, ignorant. After the battle, I found for days numerous bullet wounds which had been carefully stuffed with lint, and the plug kept in by adhesive straps, as though the surgeon desired to retain all the discharge. Considerable difference of opinion and practice prevails respecting the question of what cases should be amputated. In the inferior extremity, some sacrifice every comminuted compound fracture of the femur or tibia, while others only amputate in cases where the nerves and vessels are also destroyed. The passage of a bullet through the thigh bone, when it comminutes it badly, produces a fearful injury, with but trivial external evidence of its grave character. Hence, not a few surgeons in the army simply omit all efficient treatment in these, being lulled into a false conservatism by the trivial appearance of the bullet hole. But the internal condition is this:—the shattered and comminuted fragments of bone occupy a considerable space in the centre of the limb, creating in the surrounding tissues mortification of some portions, and intense inflammation of others. Meanwhile the orifices of the entry and exit of the ball, originally quite small, contract and prevent the free escape of pus, bone, and dead flesh from within. The result is, that the pus burrows among the muscles, the limb swells, the patient is worn out by suffering first, and discharge afterwards, and very generally dies. Still the mortality after military amputations of the thigh is so fearful that one may well stagger, being 84 per cent. at the upper third, and over 50 per cent. any where in the lower two-thirds. On the whole my experience and the careful comparison of authorities, has brought me to adopt the following rule: For gun-shot wounds through the knee or ankle joint, amputate or resect; for those of the leg and thigh which destroy the principal arteries and nerves, and at the same time shatter the bone, amputate in all cases; for those which simply fracture the femur, without much comminuting it, do not amputate; for those which are supposed to have comminuted the femur badly, without injury to the nerves and large vessels, I give the patient chloroform, and with the finger, explore the wound, thoroughly, if the bone is badly shattered, enlarge the wound to the extent of several inches and take out, at once, every loose piece of bone, and if necessary, resect the ragged extremities of the shaft; I then take pains to keep the wound well open and bide the result; by this method the terrible inflammation and burrowing of pus in the centre of the limb is prevented, a comparatively simple form of injury is produced, and the awful risks of the femoral amputation avoided; if, however, the case required transportation for days, in wagons, before any rest in a hospital could be obtained, I should certainly amputate.

I am sorry to say that I see some surgeons recklessly cutting off arms, which might as well be saved, when the same men slur over fractured thighs and perforated knee joints as though they were less serious injuries.

